# Computational and experimental identification of potential neutralizing peptides derived from human ACE2 against SARS-CoV-2 infection

**DOI:** 10.1128/jvi.01468-25

**Published:** 2026-01-30

**Authors:** Qiaobin Yao, Vidhyanand Mahase, Wangheng Hou, Ruth Cruz-Cosme, Qiyi Tang, Shaolei Teng

**Affiliations:** 1 Department of Biology, Howard University 8369, Washington, DC, USA; 2 Department of Microbiology, Howard University College of Medicine 12232, Washington, DC, USA; The Ohio State University, Columbus, Ohio, USA

**Keywords:** ACE2 mutations, SARS-CoV-2 Spike, computational saturation mutagenesis, neutralizing peptides

## Abstract

The human angiotensin-converting enzyme 2 (hACE2) is the primary receptor for the entry of severe acute respiratory syndrome coronavirus 2 (SARS-CoV-2). Some human alleles of ACE2 exhibit an improved affinity for the SARS-CoV-2 Spike protein. However, the impact of ACE2 polymorphisms on SARS-CoV-2 infection remains unclear. Our previous study predicted that G431 and S514 in the receptor-binding domain (RBD) of SARS-CoV-2 S1 domain are important for S protein stability, and that S protein residues G496 and F497 and ACE2 residues D355 and Y41 are critical for the RBD-ACE2 interaction. In this study, we explored the potential of hACE2-derived neutralizing peptides as a therapeutic strategy against SARS-CoV-2 and investigated how ACE2 polymorphisms affect RBD-ACE2 binding affinity. We applied computational saturation mutagenesis to systematically screen the binding affinity changes among all possible ACE2 missense mutations within the ACE2-Wuhan-S1 complex. Mutations at ACE2 residues D355 and Y41 were predicted to weaken binding affinity, whereas those at N330 and D30 enhanced it. We identified six ACE2 regions (19–49, 65–102, 320–333, 348–359, 378–395, and 552–563) to be vital for ACE2-RBD interaction. We synthesized peptides corresponding to these six regions and tested them using a pseudotyped viral particle system and dot blot assay. Three peptides were confirmed to bind with the S protein, and four exhibited inhibitory effects. We aligned ACE2-Wuhan-S1 and ACE2-Omicron-S1 complexes, conducted correlation analysis, and observed similar binding patterns, suggesting that these peptides also have the potential to neutralize Omicron strains.

**IMPORTANCE:**

SARS-CoV-2 continues its global spread. In this research, we identified six regions within ACE2 that are vital for interaction with the viral S receptor-binding domain and have the potential to neutralize SARS-CoV-2 infection. Among the six peptides derived from ACE2, three were confirmed to bind with the S protein of the Wuhan strain, and four exhibited inhibitory effects on the Wuhan strain SARS-CoV-2. We also found ACE2 residues D355 and Y41 as weakening affinity, and N330 and D30 as enhancing it. We also aligned this complex with the ACE2-Omicron-S1 complex, performed correlation analyses, and compared their patterns of stability changes upon mutations and obtained similar results, indicating that these peptides may also be effective against Omicron variants. These results provide insight into the role of ACE2 polymorphism in viral entry and suggest that hACE2-derived peptides may offer a promising therapeutic strategy against SARS-CoV-2, demonstrating strong consistency between our computational predictions and experimental outcomes.

## INTRODUCTION

The COVID-19 pandemic, caused by the severe acute respiratory syndrome coronavirus 2 (SARS-CoV-2), has led to unprecedented global health and economic crises (1–4). The World Health Organization (WHO) reported more than 778 million confirmed cases and nearly 7 million deaths due to SARS-CoV-2 infection as of July 2025 (5). SARS-CoV-2 is the seventh identified human coronavirus. Coronaviruses (CoVs) are a group of large and enveloped RNA viruses carrying a single-stranded positive-sense RNA genome (6). The S glycoprotein facilitates the initial stages of viral entry, including attachment to the host cell membrane and membrane fusion. The host proteases cleave the S protein into two subunits, S1 and S2. The S1 subunit is accountable for receptor angiotensin-converting enzyme 2 (ACE2) recognition, while the S2 subunit is responsible for membrane fusion (7, 8). Spike-ACE2 interaction is required for the infection of SARS-CoV-2, so we hypothesize that blocking the interaction will effectively prevent and treat SARS-CoV-2-caused disease.

There exist two major types of peptides that have been reported to be effective in preventing SARS-CoV-2 infection: ACE2-derived (9) and spike-derived (10). Peptide inhibitors derived from ACE2 with high affinity for SARS-CoV-2 could potentially address the challenges associated with antibody-based therapies, including issues in manufacturing, distribution, and drug delivery. In fact, ACE2 serves as a common receptor for several coronaviruses, positioning it as an attractive target for developing broad-spectrum coronavirus therapies that can resist viral mutations and enhance preparedness for future pandemics (11). And we hypothesize that ACE2-derived peptides not only have anti-SARS-CoV-2 activity but also present low or no toxicity.

Previous studies have primarily focused on characterizing the SARS-CoV-2 Spike-ACE2 interaction through molecular simulations and structural analyses or developing ACE2-mimicking peptides as therapeutic candidates. While these approaches have identified key binding residues, they often lack a systematic strategy for optimizing peptide sequences to improve therapeutic efficacy (12, 13). Given the lack of systematic optimization in prior approaches, computational tools can offer a rational approach to peptide design by identifying optimal modification sites to enhance stability and reduce degradation (14, 15).

In this study, we combined computational and experimental approaches to develop anti-SARS-CoV-2 peptides. Six ACE2-derived peptides were designed and tested with a pseudotyped viral system, demonstrating that these peptides effectively reduced viral entry in a cell culture system. This approach enables a more precise and efficient peptide design strategy, overcoming key limitations in existing ACE2-based therapeutics and offering a promising framework for the development of stable and effective antiviral peptides.

## RESULTS

### Identification of ACE2 regions that bind to SARS-CoV-2 spike protein

**Fig 1:**
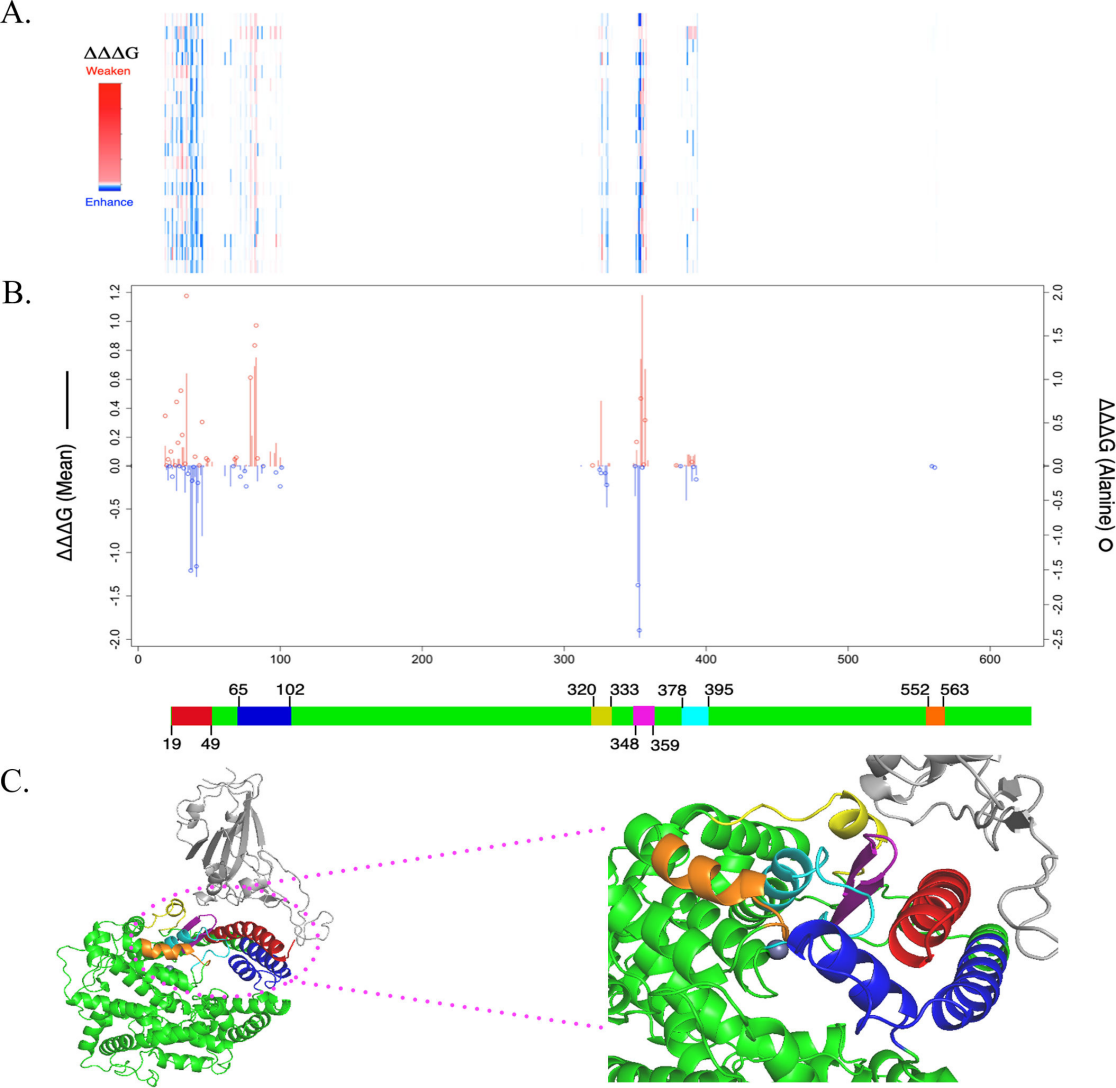
Effects of residues and mutations on ACE2-Wuhan-S1 interaction. (**A**) Heatmap showing predicted ΔΔG values for all mutations across residues (x-axis: amino acid position). The color scale ranges from −5 to +80 kcal/mol, with blue to light blue indicating stabilizing effects, white neutral, and pink to red indicating increasing destabilization. The same residue positions are shown in panel B. (**B**) Line chart summarizing mean ΔΔG values (left Y-axis, solid line) and ΔΔG of alanine substitutions (right Y-axis, circles) across ACE2 residues. Red indicates decreased binding affinity (destabilizing), and blue indicates increased binding affinity (stabilizing). (**C**) Six peptide regions are shown in PyMol images. ACE2 is shown in green. Wuhan-Hu-S-1 is shown in gray. Region 1 (19–49): Red; Region 2 (65–102): Blue; Region 3 (320–333): Yellow; Region 4 (348–359): Purple; Region 5 (378–395): Cyan; Region 6 (552–563): Orange.

To generate the required information supporting the development of ACE2-derived neutralizing peptides against SARS-CoV-2, we computationally evaluated the effects of 113,324 non-redundant missense mutations in the ACE2 protein within the ACE2-Wuhan-S1 complex (PDB: 6LZG). Specifically, we calculated the binding affinity changes (ΔΔΔG) resulting from these mutations. A heatmap of ΔΔΔG values is shown in Fig. 1A, while Fig. 1B presents the mean values of ΔΔΔG at each ACE2 residue position, along with the ΔΔΔG value of substitutions to alanine.

Our analysis identified six key ACE2 regions—residues 19–49, 65–102, 320–333, 348–359, 378–395, 552–563—that are associated with significant changes in binding affinity. These regions appear to play a critical role in stabilizing the interaction between the human ACE2 and the SARS-CoV-2 S1 protein. Given their importance in receptor-virus binding, these ACE2 regions represent promising candidates for the design of ACE2-derived peptides capable of competitively inhibiting SARS-CoV-2 by mimicking native ACE2 and blocking the spike protein’s receptor-binding domain (RBD). Such peptides may offer an effective therapeutic strategy against SARS-CoV-2 infection.

### Effects of ACE2 mutations on ACE2-Wuhan-S1 interaction

**Fig 2:**
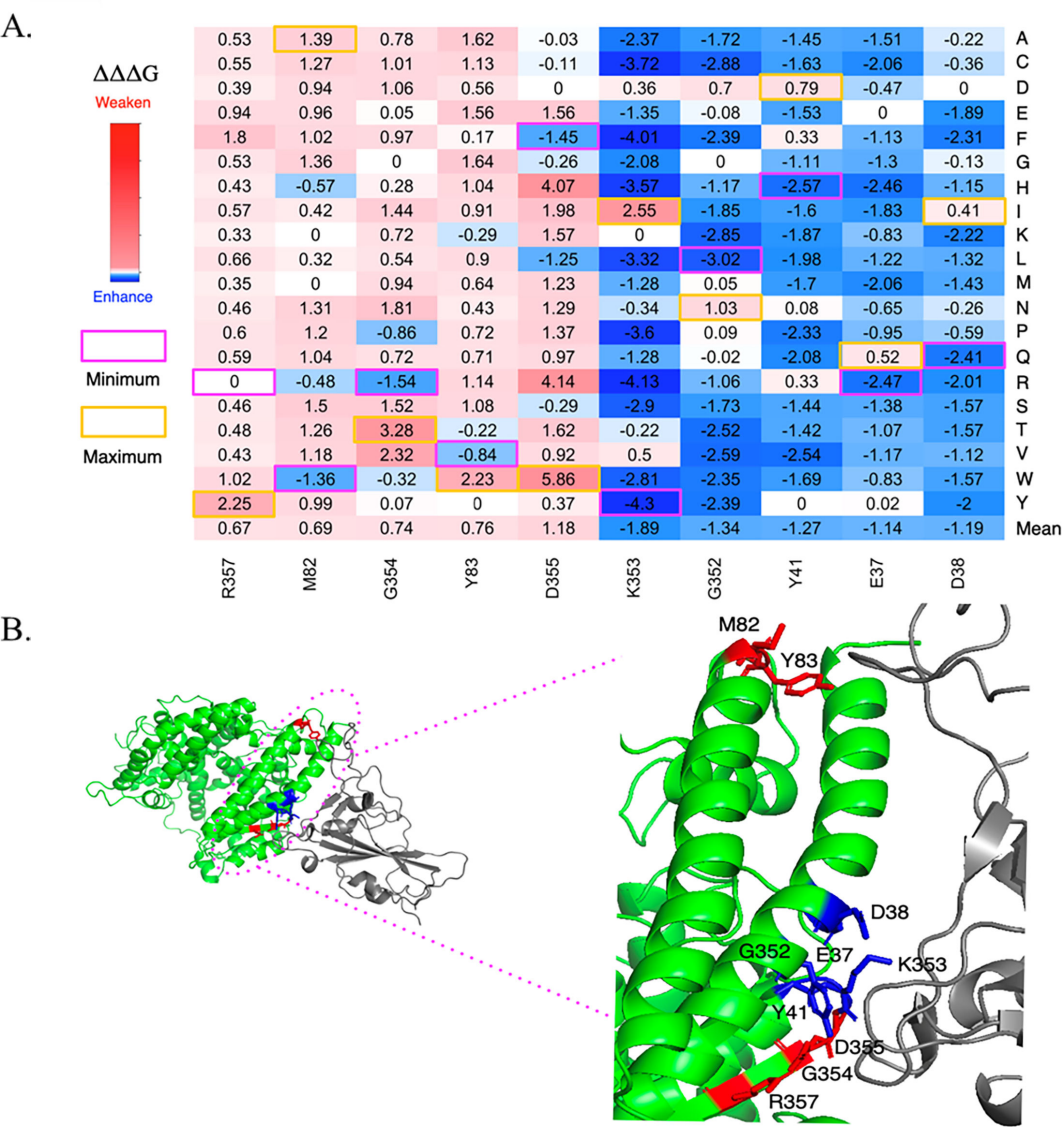
Key residues that affect ACE2-Wuhan-S1 binding affinity. (**A**) Heatmaps are shown as maximum values (yellow) and minimum (magenta), with intermediate shades representing gradual changes. The color scale ranges from blue (ΔΔΔG > -5 kcal/mol), passing through dodger blue, white (near-neutral ΔΔΔG ≈ 0 kcal/mol), and pink (ΔΔΔG < 6 kcal/mol). Numerical values corresponding to the color gradient are indicated on the color bar for clarity. (**B**) Key residues are shown in blue or red in PyMol images. ACE2 is shown in green. Wuhan-S1 is shown in gray.

To determine how ACE2 mutations affect the ACE2-Wuhan-S1 interaction, we selected key ACE2 residues whose alterations significantly influence binding affinity. Based on the mean values of ΔΔΔG, five residues—R357, M82, G354, Y83, and D355—were found to enhance binding affinity, while five other residues—K353, G352, Y41, E37, and D38—reduced the binding affinity. Among these, residue K353 showed the strongest enhancing effect (ΔΔΔG mean = −1.89 kcal/mol), with the K353Y mutation exhibiting the greatest reduction in binding free energy (ΔΔΔG =−4.3 kcal/mol), indicating a significant increase in ACE2-Wuhan-S1 binding. Conversely, residue D355 had the strongest weakening effect (ΔΔΔG mean = 1.18 kcal/mol), with the D355W mutation yielding the highest ΔΔΔG value at 5.86 kcal/mol, reflecting weakened interaction (Fig. 2A, Table 1).

**TABLE 1:** Effects of key mutations on ACE2-SARS-CoV-2 S interaction

ACE2-Wuhan-S1 complex			ACE2-Omicron-S1 complex		
Variant	ΔΔΔG	rs ID	Variant	ΔΔΔG	rs ID
R357Y	2.25	NA^*a*^	D355F	5.09	NA
M82A	1.39	NA	Y41G	2.94	NA
G354T	3.28	NA	Y83G	1.88	NA
Y83W	2.23	NA	G354P	4.88	NA
D355W	5.86	NA	E35K	3.35	rs1348114695
K353Y	−4.3	NA	H34W	−3.39	NA
G352L	−3.02	NA	L39E	−2.2	NA
Y41H	−2.57	rs768024356	F72W	−1.84	NA
E37R	−2.47	NA	K31L	−2.17	NA
D38Q	−2.41	NA	D30L	−2.43	NA
K26R	0.01	rs4646116	K26R	−0.02	rs4646116
K341R	0	rs138390800	K341R	0.07	rs138390800
S19P	0.88	rs73635825	S19P	0.4	rs73635825
E37K	−0.83	rs146676783	E37K	0.33	rs146676783
M82I	0.42	rs766996587	M82I	0.36	rs766996587

^*a*^ N/A, these mutations are not available in the dbSNP database.

As shown in Fig. 2B, all those residues are located at the binding interfaces of the ACE2-Wuhan-S1 complex and participate in critical interaction network. Notably, residues R357, G354, D355, K353, Y41, E37, and D38 have been previously reported as key residues in both ACE2-SARS-CoV-2 S RBD and ACE2-SARS-CoV S RBD interfaces (16, 17). D30, K31, and K353 interact with the K417, E484, and L452 of SARS-CoV-2 S protein, and the mutations on those residues may facilitate antibody escape. Y41 interacts with the N501Y of SARS-CoV-2 S protein, and both the ACE2 Y41 mutations and the S N501Y mutation have been shown to enhance ACE2-Wuhan-S1 binding affinity (18, 19).

Moreover, we found that the residues with significant effects on binding affinity are clustered within these specific regions (Fig. 1 and 2). E37, D38, and Y41 are located in the 1st region (19–49); M82 and Y83 in the 2nd region (65–102); and G352, K353, G354, D355, and R357 in the 4th region. In addition, we investigated the binding energy change induced by human genetic variations. The common variants, E37K and G352V, can enhance the ACE2-Wuhan-S1 interaction. In contrast, variants M82I and D355N can weaken the ACE2-Wuhan-S1 interaction.

### Experimental validation of hACE2-derived peptides binding to SARS-CoV-2 Spike protein

**Fig 3:**
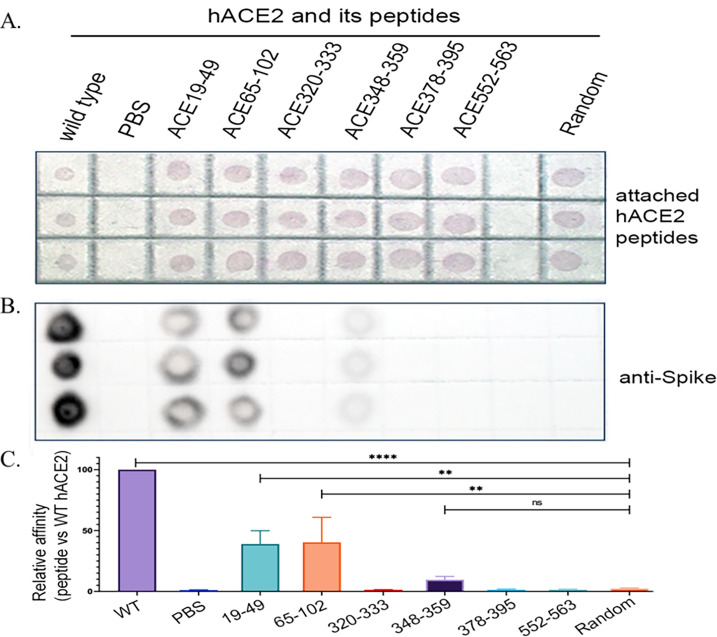
*In vitro* binding assay followed by a dot blot analysis. (**A**) Each peptide was spotted onto an NC membrane (red); (**B**) detection of bound S protein was performed by dot blot assay; and (**C**) quantitative analysis. Statistical significance is indicated as follows: ns, not significant (*P* ≥ 0.05); **P < 0.01; ****P < 0.0001.

To confirm the binding affinity of the six human angiotensin-converting enzyme 2 (hACE2)-derived peptides to the Spike protein of SARS-CoV-2, we conducted an *in vitro* binding assay followed by a dot blot analysis. First, each peptide dissolved in RPMI-1640 was spotted onto an NC membrane at equal amounts. After naturally drying, the membrane was visualized to capture the red spots as shown in Fig. 3A, showing an equal amount of peptides spotted onto the membrane. The membrane was then fixed and subsequently incubated with an S protein-containing solution. Following three washes with PBS-T, the membrane was probed with HRP-labeled anti-Spike antibody. Detection of bound S protein was performed by dot blot assay. The results showed strong binding of the S protein to peptides ACE19–49 and ACE65–102 and weak affinity with ACE348–359 (Fig. 3B). The remaining peptides showed no detectable binding. Quantitative analysis revealed that the relative binding affinities of ACE19–49 and ACE65–102 were significantly higher than that of a random control peptide (Fig. 3C). These findings demonstrate that the ACE2-S binding predicted by the computational modeling requires experimental confirmation.

### ACE2-derived peptides significantly inhibit SARS-CoV-2 infection

**Fig 4:**
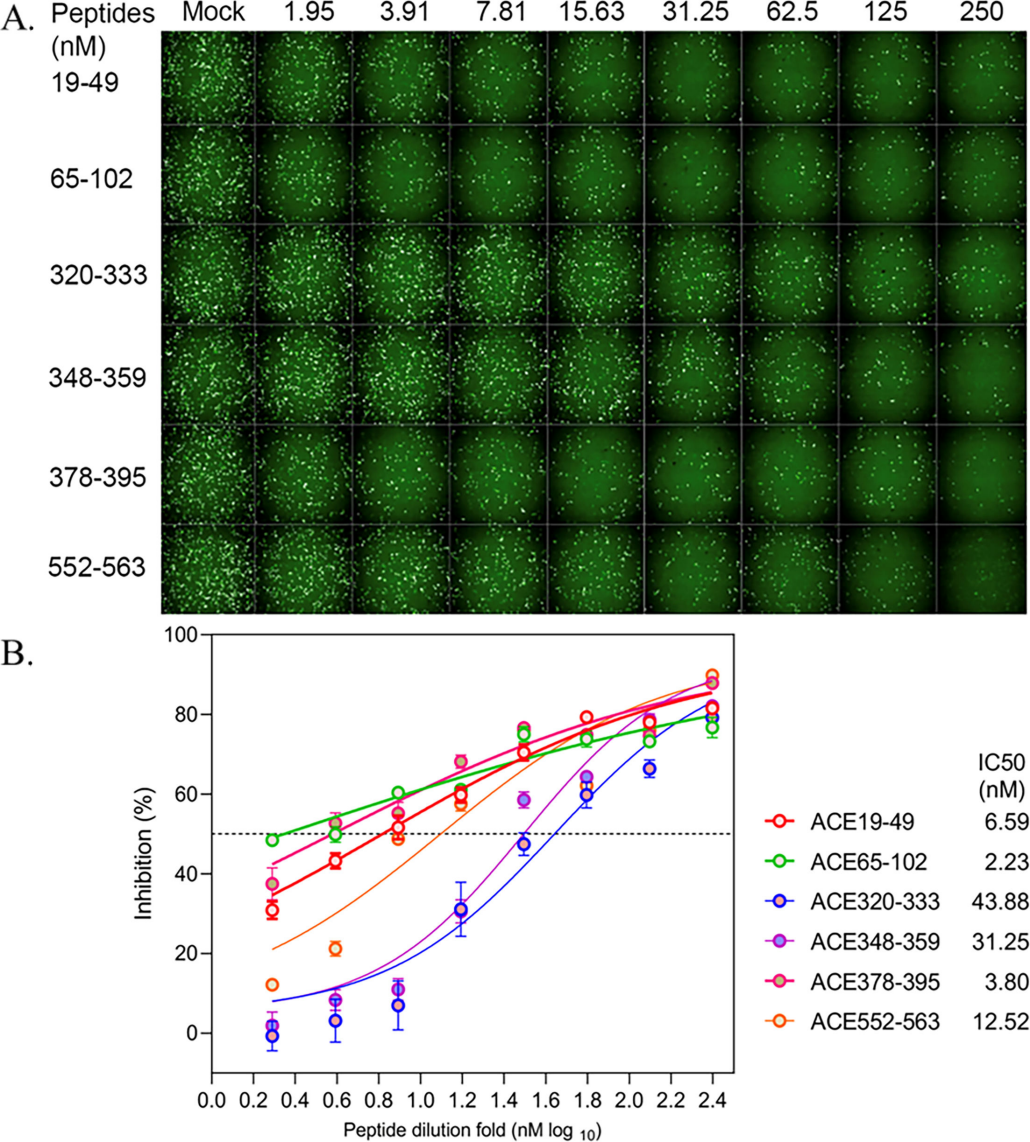
The inhibitory effects of the peptides: using the pseudotyped viral particle system to evaluate the inhibitory effects of the peptides in a cell culture model. (**A**) Representative fluorescence images showing dose-dependent inhibition of infection by ACE2 peptides (1.95–250 nM). (**B**) Dose-response curves and IC₅₀ values of each peptide, showing the strongest inhibition by ACE65–102 and ACE378–395.

To evaluate whether hACE2-derived peptides inhibit SARS-CoV-2 infection, we employed a pseudotyped viral particle system to circumvent the need for a BSL-3 facility required for live SARS-CoV-2. This system allowed us to assess the inhibitory effects of the peptides in a cell culture model. As shown in Fig. 4, a twofold serial dilution of each peptide was mixed with the pseudotyped SARS-CoV-2 particle and used to infect BHK-hACE2 cells. All six hACE2-derived peptides significantly reduced pseudotyped SARS-CoV-2 infection in a dose-dependent manner. Notably, the two peptides with the strongest spike protein-binding affinities exhibited the most potent inhibitory effects, with IC50 values of 6.59 nM and 2.23 nM, respectively. Interestingly, the peptide of ACE378–395, despite its low binding affinity, also demonstrated strong inhibition of viral infection. This discrepancy suggests that additional mechanisms may contribute to its inhibitory effect and warrants further investigation. These findings demonstrate that the computationally predicted hACE2-derived peptides can effectively attenuate SARS-CoV-2 infection in hACE2-expressing cells. Moreover, our established pseudotyped virus system provides a valuable platform for studying the inhibitory effects of potential therapeutic agents against SARS-CoV-2.

### Comparison of ACE2-Wuhan-S1 and ACE2-Omicron-S1 complexes

**Fig 5:**
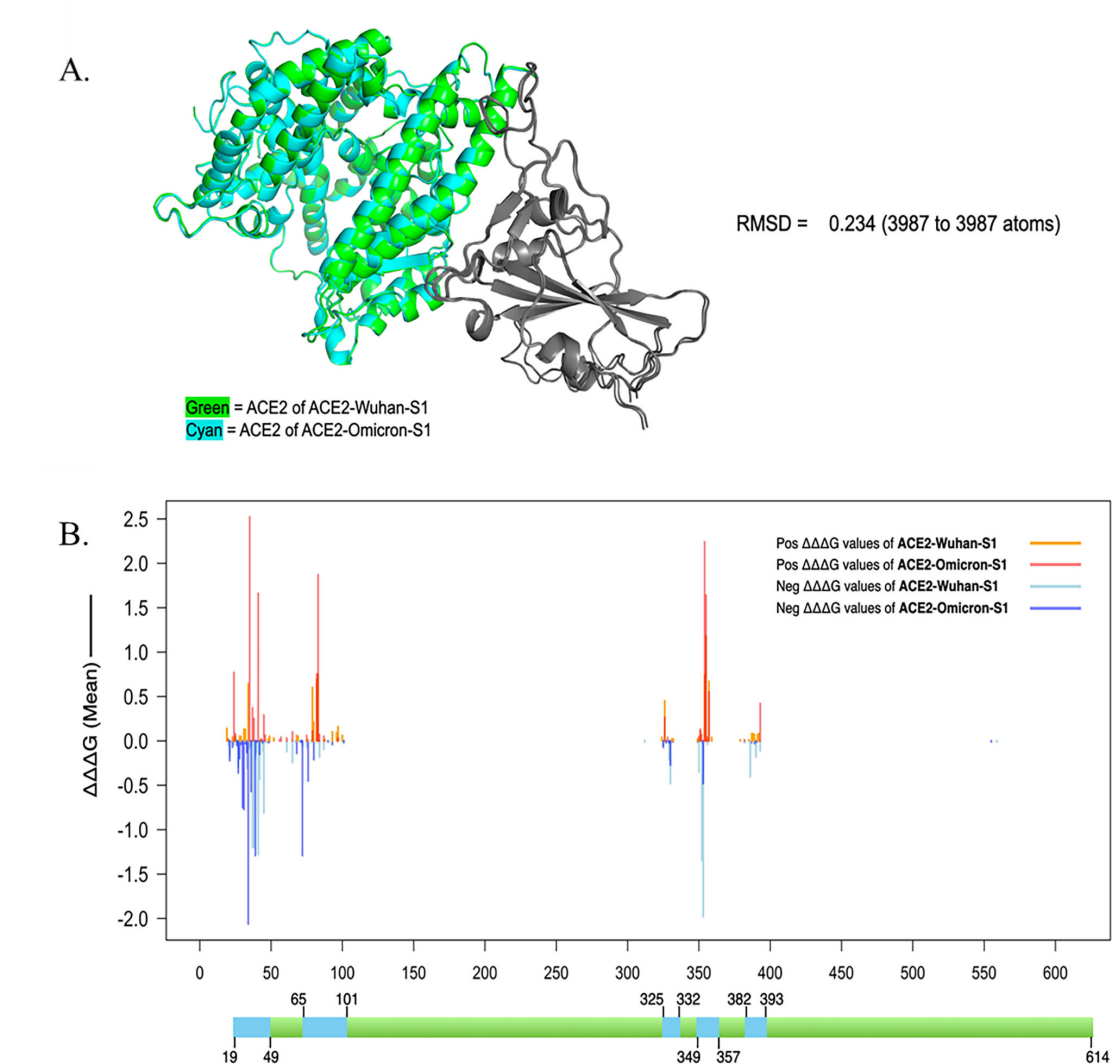
Comparison of ACE2-Wuhan-S1 and ACE2-Omicron-S1 complexes. (**A**) Structural alignment of ACE2-Wuhan-S1 and ACE2-Omicron-S1 complexes. (**B**) Combined line chart of the mean binding affinity ΔΔΔG.

To investigate whether these potential ACE2-neutralizing peptides can also be applicable to the ACE2-Omicron-S1 complex, we performed structural alignment of the ACE2 chains of both the ACE2-Wuhan-S1 and ACE2-Omicron-S1 complexes (Fig. 5A) and compared ΔΔΔG values of ACE2 mutations in both complexes (Fig. 5B). The ACE2-Omicron-S1 complex used in this analysis corresponds to the Omicron B.1.1.529 (BA.1) subvariant, as determined from the primary citation of the structural data set (PDB ID: 7WBP) (20). Comparing the root-mean-squared deviation (RMSD) of corresponding atoms in two proteins is a standard measure of their similarity. Lower RMSD values indicate greater similarity. The RMSD value between the two complexes is 0.234 (3,987–3,987 atoms), which indicates a high degree of structural similarity between ACE2 conformations in both complexes (Fig. 5A). Structural alignment and correlation analysis revealed consistent patterns in how ACE2 mutations affect binding in both ACE2-Wuhan-S1 and ACE2-Omicron-S1 complexes. The mean values of ΔΔΔG at each ACE2 residue position in both complexes are shown in the line charts of Fig. 5B. As shown in the combined line chart, although some top residues differed, five ACE2 regions with potential as neutralizing peptides overlapped almost completely between the two complexes.

## DISCUSSION

SARS-CoV-2 infection is initiated by the binding of the viral spike protein to the host receptor ACE2, a critical step that governs viral entry and pathogenesis (7). Genetic variation in ACE2 has been reported to alter binding affinity with the spike RBD (21–23), yet its implications for viral susceptibility and therapeutic intervention remain incompletely understood. ACE2-derived peptides offer the potential for broad-spectrum inhibition of coronaviruses (12, 13) while minimizing issues associated with antibody-based therapies (14, 15). Our study systematically investigated ACE2 polymorphisms and designed ACE2-derived peptides to evaluate their capacity to interfere with SARS-CoV-2 entry, which bridged computational predictions with experimental validation.

This study focuses on investigating the potential of ACE2-based peptides and analyzing key residues in ACE2 proteins that are important for their interaction with the S protein, based on the binding affinity changes caused by ACE2 missense mutations. We used the ACE2-Wuhan-S1 complex as a reference to identify potential peptide candidates. First, a computational saturation mutagenesis aimed at discovering ACE2-derived peptides capable of neutralizing SARS-CoV-2 identified five regions within the ACE2 protein (residues 19–49, 65–101, 325–332, 349–357, and 382–393) that are highly likely to interact with the S protein. Next, the affinities of these peptides to the spike protein were tested *in vitro*, and three out of five showed higher affinities. Additionally, we used a pseudotyped viral particle system to test the peptides’ effects on the infection of SARS-CoV-2, and the result showed that the ACE2-derived peptides significantly attenuated the viral infection in hACE2-complemented cells, suggesting the potential of computationally identified peptides in reducing the severity of SARS-CoV-2 infection.

Notably, the peptide ACE378–395 strongly inhibited pseudoviral infection despite a relatively low binding affinity *in vitro*. This discrepancy suggests that mechanisms beyond direct binding, such as conformational blocking (23), interference with spike trimerization (24), or allosteric effects on the S-ACE2 interface, may contribute to its inhibitory efficacy, warranting further investigation.

Furthermore, we performed a structural alignment between the ACE2-Wuhan-S1 and ACE2-Omicron-S1 complexes to assess variations in their binding interfaces. To quantitatively compare the binding affinity between these two complexes, we conducted a correlation analysis, followed by a comparative examination of line charts illustrating the binding trends. Our results demonstrated a high degree of similarity between the two complexes, suggesting that the identified peptides may also have the potential to neutralize SARS-CoV-2 Omicron variants.

We analyzed the human ACE2 genetic variants, as these polymorphisms can influence ACE2 structure, stability, expression, and its binding affinity to the SARS-CoV-2 spike protein, thereby affecting individual susceptibility and disease severity. The prevalence of ACE2 genetic variants differs across populations, potentially contributing to disparities in COVID-19 outcomes (25, 26). Notably, variants such as rs4646116 (K26R) and rs138390800 (K341R) are associated with reduced protein stability and are more common in certain populations (27). Several known ACE2 variants, including rs73635825 (S19P), rs146676783 (E37K), and rs766996587 (M82I), showed distinct effects across viral strains (Table 1). For instance, the M82I and S19P variants decreased binding affinity in the Omicron complex but had minimal impact in the Wuhan complex. Conversely, the E37K variant increased binding in the Wuhan interaction (ΔΔΔG = −1.83 kcal/mol) but reduced it in Omicron (ΔΔΔG = 0.22 kcal/mol), emphasizing the variant-specific nature of ACE2-spike interactions. We further examined the impact of specific ACE2 mutations on spike binding. Within the ACE2-Wuhan-S1 complex, mutations at D355, Y83, and G354 significantly weakened binding affinity, while K353, G352, and Y41 enhanced it (Fig. 2A). Interestingly, the Y41 mutation exhibited opposite effects between variants, enhancing ACE2-Wuhan-S1 binding (ΔΔΔG = −1.27 kcal/mol) but weakening binding in the ACE2-Omicron-S1 complex (ΔΔΔG = 1.88 kcal/mol).

This study investigates the development of targeted therapeutic strategies by identifying key residues and mutations that affect the ACE2 protein’s binding with the SARS-CoV-2 S1 protein. In particular, we highlight neutralizing peptides that can block this critical interaction. Our computational analyses offer valuable insights into the systemic effects of ACE2 mutations on binding, supporting the rational design of effective treatments and vaccines to help mitigate the ongoing impact of COVID-19.

## MATERIALS AND METHODS

### Structure preparation

To investigate the impact of mutations on the interaction between Omicron-S (Omicron B.1.1.529 (BA.1) subvariant) and hACE2, we conducted a comparison between ACE2-Omicron-S1 (PDB ID: 7WBP), a crystal structure that illustrates the binding of Omicron-S to ACE2, and ACE2-Wuhan-S1 (PDB ID: 6LZG), which reveals the configuration of RBD and RBM proteins that facilitate viral entry. The structures were acquired from the Protein Data Bank (28).

### Mutation collection

To assess the impact of mutations on proteins, we applied computational saturation mutagenesis to mutate all residues in the corresponding structures to all 19 other amino acid types. This approach entails introducing mutations at these specific locations to incorporate all conceivable amino acids. Subsequently, the resulting variants are examined to identify any potential alterations in protein stability or interactions with other proteins (29). Specifically, we employed an in-house Perl script to produce the list of all possible mutations in each residue. Then, we used the lists in the FoldX (30) program to simulate the effect of the mutation on the intricate protein structure. We collected ACE2 genetic variations in different populations from two data sources: gnomAD v4.3 (31) and HGMD (32).

### Energy calculations

Before doing any energy calculations, all protein structures were fixed using the “RepairPDB” command on the command line interface on the FoldX official website. This command operates by altering certain residues to reduce the overall free energy of the protein structure. The FoldX program was utilized for interaction analysis and computing saturation mutagenesis, total energy calculations, Van der Waals interactions, hydrogen bonding, and other energy correlations.

The calculation of binding energy change (ΔΔΔG) was performed using FoldX for each mutation. The “AnalyseComplex” command was utilized to determine the free folding energy change, specifically the binding energy change between the contacts. We calculated the ΔΔG(binding) by breaking down each protein and analyzing its energies. To get the change in binding free energy (ΔΔΔG), we subtract the ΔΔG(binding) value from the total energy of the protein complex, specifically from the wild-type complexes. The mathematical equation provides the value of the change in the binding free energy (ΔΔΔG) between the mutant structure and the wild-type structure.


(1) ΔΔG(binding)=ΔG(folding:AB)−ΔG(folding:A)−ΔG(folding:B)

(2) ΔΔΔG(binding)=ΔΔG(binding)MUT−ΔΔG(binding)WT

A negative value of ΔΔΔG indicates an increased binding affinity, whereas a positive value denotes a decrease in binding affinity. Using the obtained ΔΔΔG values, we employed the R program to create heatmaps and line graphs (33).

### Structural alignments and correlation analysis

We also used PyMOL (34) to do the Structural Alignments between ACE2-Wuhan-S1 (PDB ID: 6LZG) and ACE2-Omicron-S1 (PDB ID: 7WBP) by using the PyMOL software’s “fetch and align” commands, as well as display their key mutations, resulting in the generation of visual representations of 3D structures. Also, we did the correlation analysis to compare the dddG values of those two structures by using the R package (33). Also, R was used to generate graphs and perform analysis of variance test and *t-*test in statistical comparisons of energy changes. According to the results, we found six potential peptides in different regions: (i) Peptide 1: STIEEQAKTFLDKFNHEAEDLFYQSSLASWN, from the regions 19 to 49; (ii) Peptide 2: NMNNAGDKWSAFLKEQSTLAQMYPLQEIQNLTVKLQLQALQQ, from the regions 65 to 102; (iii) Peptide 3: LPNMTQGFWENSML, from the regions 320 to 333; (iv) Peptide 4: AWDLGKGDFRIL, from the regions 348 to 359; (v) Peptide 5: HIQYDMAYAAQPFLLRNG, from the regions 378 to 395; (vi) Peptide 6: QKLFNMLRLGKS, from the regions 552 to 563.

### Binding assays of spike-hACE2 and spike-hACE2-derived peptides

A dot blot assay was performed to evaluate the binding of hACE2 and its derived peptides to the SARS-CoV-2 Spike protein. Briefly, hACE2 and the peptides (synthesized and purified by Sino Biological and Sangon Biotech, Shanghai) were dissolved in RPMI-1640 medium (no serum) and spotted onto a nitrocellulose membrane (12) at 0.5 μg per dot. After air drying, an initial photograph was taken to capture the red color of phenol red in RPMI-1640 (Fig. 3A). The membrane was fixed with 4% paraformaldehyde at room temperature for 10 min, washed with PBS-T (PBS containing 0.05% Tween-20), and air-dried at room temperature. Subsequently, the membrane was incubated with PBS containing 5% skim milk for 1 h at room temperature with gentle shaking. After three washes with PBS-T, the membrane was incubated with the Spike protein solution (0.1 mg/mL) for 1 hour at room temperature with gentle shaking. After three washes with PBS-T, the membrane was treated with an anti-spike antibody conjugated with horseradish peroxidase (HRP) (2F9-HRP, New England Biolabs). Positive signals were detected using the ChemiDoc MP System (Bio-Rad) with the SuperSignal West Femto Maximum Sensitivity Substrate. The grayscale intensities of the dots were quantified using ImageJ software.

### Inhibitory effects of the hACE2-derived peptides on SARS-CoV-2 infection

A pseudotyped viral particle system was used to avoid using the actual viral particle which needs a BSL-3 facility. The pseudotyped ΔG-GFP (G*ΔG-GFP) rVSVw/pCAGGS-G-Kan system was purchased from Kerafast Company (https://www.kerafast.com/productgroup/171/ pseudotyped-g-gfp-gg-gfp-rvsv). ΔG-GFP is a modified form of vesicular stomatitis virus (rVSV) that is limited in its ability to replicate, and the infectivity of these pseudotyped viruses is limited to a single round of replication: they can be handled using standard BSL-2 containment practices.

The spike gene of the Wuhan-Hu-1 strain (GenBank: MN908947) was codon-optimized for expression in human cells and cloned into the eukaryotic expression plasmid pCAG to create pCAG-nCoV-S. The plasmid pCAG-nCoV-S was introduced into Vero-E6 cells and incubated for 48 h. Subsequently, the VSVdG-EGFP-G virus (35) was inoculated to the cells expressing SARS-CoV-2 spike protein for 1 h. Then, VSVdG-EGFP-G virus-containing supernatant was replaced with medium containing anti-VSV-G rat serum that blocks the infection of residual rVSVdG-G. The supernatant was collected at 24 h post-rVSVdG-G infection and centrifuged, and filtered (0.45-μm pore size) to remove cell debris, aliquoted, and stored at −80°C for use. The synthetic hACE2-derived peptides were diluted in a continuous gradient twofold from 250 nM to 1.95 nM and mixed with VSV-SARS-CoV-2-S virus (MOI = 0.05) and incubated at 37°C for 1 h. The mixtures were added to BHK21-hACE2 cells for 12 h. Fluorescent assays were performed to visualize the infected cells (GFP).

## Data Availability

The computational data supporting the findings of this study are provided within the main text. Experimental data are available from the corresponding authors upon request.

## References

[1] Cocking E, Daher J, Alabbood M. 2025. New-onset diabetes mellitus post COVID-19 infection: a systematic review and meta-analysis. Diabetes Res Clin Pract 227:112417. doi:10.1016/j.diabres.2025.112417 40803509

[2] Gil AM, Barahona-Correa J, Flórez JB, Fernández-Ávila DG, Cucunubá ZM. 2025. Risk of new onset of immune-mediated diseases after SARS-CoV-2 infection: a systematic review and meta-analysis. Semin Arthritis Rheum 74:152805. doi:10.1016/j.semarthrit.2025.152805 40812100

[3] Pierce M, Banwell E, Gillibrand S, Panayiotou M, Qualter P, Munford L, Demkowicz O. 2025. Changes in mental health of adolescents during three months of the COVID-19 pandemic: longitudinal study. BJPsych Open 11:e177. doi:10.1192/bjo.2025.10801 40814699 PMC12451728

[4] Usta T, Biberoğlu S, İpekci A, İkizceli İ, Çakmak F, Akdeniz YS, Baktıroğlu G, Özkan S. 2025. Effects of the COVID-19 pandemic on anxiety and depression among medical interns. West J Emerg Med 26:795–803. doi:10.5811/westjem.38455 40795006 PMC12342501

[5] World Health Organization. July, 2025. WHO Coronavirus Disease (COVID-19) Dashboard. Available from: https://covid19.who.int

[6] Lu G, Liu D. 2012. SARS-like virus in the Middle East: a truly bat-related coronavirus causing human diseases. Protein Cell 3:803–805. doi:10.1007/s13238-012-2811-1 23143870 PMC4875465

[7] Jackson CB, Farzan M, Chen B, Choe H. 2022. Mechanisms of SARS-CoV-2 entry into cells. Nat Rev Mol Cell Biol 23:3–20. doi:10.1038/s41580-021-00418-x 34611326 PMC8491763

[8] Yang H, Rao Z. 2021. Structural biology of SARS-CoV-2 and implications for therapeutic development. Nat Rev Microbiol 19:685–700. doi:10.1038/s41579-021-00630-8 34535791 PMC8447893

[9] Larue RC, Xing E, Kenney AD, Zhang Y, Tuazon JA, Li J, Yount JS, Li P-K, Sharma A. 2021. Rationally designed ACE2-derived peptides inhibit SARS-CoV-2. Bioconjug Chem 32:215–223. doi:10.1021/acs.bioconjchem.0c00664 33356169 PMC7784661

[10] Pesce E, Manfrini N, Cordiglieri C, Santi S, Bandera A, Gobbini A, Gruarin P, Favalli A, Bombaci M, Cuomo A, Collino F, Cricrì G, Ungaro R, Lombardi A, Mangioni D, Muscatello A, Aliberti S, Blasi F, Gori A, Abrignani S, De Francesco R, Biffo S, Grifantini R. 2021. Exosomes recovered from the plasma of COVID-19 patients expose SARS-CoV-2 spike-derived fragments and contribute to the adaptive immune response. Front Immunol 12:785941. doi:10.3389/fimmu.2021.785941 35111156 PMC8801440

[11] Chen Y, Zhang Y-N, Yan R, Wang G, Zhang Y, Zhang Z-R, Li Y, Ou J, Chu W, Liang Z, Wang Y, Chen Y-L, Chen G, Wang Q, Zhou Q, Zhang B, Wang C. 2021. ACE2-targeting monoclonal antibody as potent and broad-spectrum coronavirus blocker. Sig Transduct Target Ther 6:315. doi:10.1038/s41392-021-00740-y PMC8385704 34433803

[12] Boshah H, Samkari F, Valle-Pérez AU, Alsawaf SM, Aldoukhi AH, Bilalis P, Alshehri SA, Susapto HH, Hauser CAE. 2023. Evaluation of potential peptide-based inhibitors against SARS-CoV-2 and variants of concern. Biomed Res Int 2023:3892370. doi:10.1155/2023/3892370 37869628 PMC10589072

[13] Sarto C, Florez-Rueda S, Arrar M, Hackenberger CPR, Lauster D, Di Lella S. 2022. Atomistic insight into the essential binding event of ACE2-derived peptides to the SARS-CoV-2 spike protein. Biol Chem 403:615–624. doi:10.1515/hsz-2021-0426 35357791

[14] Paul S, Nadendla S, Sobhia ME. 2022. Identification of potential ACE2-derived peptide mimetics in SARS-CoV-2 omicron variant therapeutics using computational approaches. J Phys Chem Lett 13:7420–7428. doi:10.1021/acs.jpclett.2c01155 35929665

[15] Sarma S, Herrera SM, Xiao X, Hudalla GA, Hall CK. 2022. Computational design and experimental validation of ACE2-derived peptides as SARS-CoV-2 receptor binding domain inhibitors. J Phys Chem B 126:8129–8139. doi:10.1021/acs.jpcb.2c03918 36219223

[16] Suryamohan K, Diwanji D, Stawiski EW, Gupta R, Miersch S, Liu J, Chen C, Jiang Y-P, Fellouse FA, Sathirapongsasuti JF, et al.. 2021. Human ACE2 receptor polymorphisms and altered susceptibility to SARS-CoV-2. Commun Biol 4:475. doi:10.1038/s42003-021-02030-3 33846513 PMC8041869

[17] Teng S, Sobitan A, Rhoades R, Liu D, Tang Q. 2021. Systemic effects of missense mutations on SARS-CoV-2 spike glycoprotein stability and receptor-binding affinity. Brief Bioinform 22:1239–1253. doi:10.1093/bib/bbaa233 33006605 PMC7665319

[18] Jawad B, Adhikari P, Podgornik R, Ching WY. 2021. Key interacting residues between RBD of SARS-CoV-2 and ACE2 receptor: combination of molecular dynamics simulation and density functional calculation. J Chem Inf Model 61:4425–4441. doi:10.1021/acs.jcim.1c00560 34428371

[19] Oliveira EH, Monteleone-Cassiano AC, Tavares L, Santos JC, Lima TM, Gomes GF, Tanaka PP, Monteiro CJ, Munuera M, Batah SS, Fabro AT, Faça VM, Masson AP, Donadi EA, Dametto M, Bonacin R, Martins RB Jr, Neto EA, daSilva LLP, Cunha TM, Passos GA. 2024. A mimetic peptide of ACE2 protects against SARS-CoV-2 infection and decreases pulmonary inflammation related to COVID-19. Antiviral Res 229:105968. doi:10.1016/j.antiviral.2024.105968 39004311

[20] Han P, Li L, Liu S, Wang Q, Zhang D, Xu Z, Han P, Li X, Peng Q, Su C, Huang B, Li D, Zhang R, Tian M, Fu L, Gao Y, Zhao X, Liu K, Qi J, Gao GF, Wang P. 2022. Receptor binding and complex structures of human ACE2 to spike RBD from omicron and delta SARS-CoV-2. Cell 185:630–640. doi:10.1016/j.cell.2022.01.001 35093192 PMC8733278

[21] Chen W, Lee J-Y, Kim J-S, Shin JS, Fung TS, Yeh J-Y, Chen Z, Zhou B, Song J-J, Go YY. 2025. Critical amino acid residues in human ACE2 for SARS-CoV-2 spike protein binding and virus entry. Microbiol Spectr 13:e0324424. doi:10.1128/spectrum.03244-24 40539804 PMC12323325

[22] MacGowan SA, Barton MI, Kutuzov M, Dushek O, van der Merwe PA, Barton GJ. 2022. Missense variants in human ACE2 strongly affect binding to SARS-CoV-2 Spike providing a mechanism for ACE2 mediated genetic risk in Covid-19: A case study in affinity predictions of interface variants. PLoS Comput Biol 18:e1009922. doi:10.1371/journal.pcbi.1009922 35235558 PMC8920257

[23] Rodriguez JA, Gonzalez J, Arboleda-Bustos CE, Mendoza N, Martinez C, Pinzon A. 2022. Computational modeling of the effect of five mutations on the structure of the ACE2 receptor and their correlation with infectivity and virulence of some emerged variants of SARS-CoV-2 suggests mechanisms of binding affinity dysregulation. Chem Biol Interact 368:110244. doi:10.1016/j.cbi.2022.110244 36336003 PMC9630301

[24] Han P, Su C, Zhang Y, Bai C, Zheng A, Qiao C, Wang Q, Niu S, Chen Q, Zhang Y, Li W, Liao H, Li J, Zhang Z, Cho H, Yang M, Rong X, Hu Y, Huang N, Yan J, Wang Q, Zhao X, Gao GF, Qi J. 2021. Molecular insights into receptor binding of recent emerging SARS-CoV-2 variants. Nat Commun 12:6103. doi:10.1038/s41467-021-26401-w 34671049 PMC8528823

[25] Ali F, Elserafy M, Alkordi MH, Amin M. 2020. ACE2 coding variants in different populations and their potential impact on SARS-CoV-2 binding affinity. Biochem Biophys Rep 24:100798. doi:10.1016/j.bbrep.2020.100798 32844124 PMC7439997

[26] Strafella C, Caputo V, Termine A, Barati S, Gambardella S, Borgiani P, Caltagirone C, Novelli G, Giardina E, Cascella R. 2020 Analysis of ACE2 genetic variability among populations highlights a possible link with COVID-19-related neurological complications. Genes (Basel) 11:741. doi:10.3390/genes11070741 32635188 PMC7397291

[27] Mahase V, Sobitan A, Rhoades R, Zhang F, Baranova A, Johnson M, Otolorin A, Tang Q, Teng S. 2022. Genetic variations affecting ACE2 protein stability in minority populations. Front Med (Lausanne) 9:1002187. doi:10.3389/fmed.2022.1002187 36388927 PMC9659633

[28] Burley SK, Berman HM, Kleywegt GJ, Markley JL, Nakamura H, Velankar S. 2017. Protein Data Bank (PDB): the single global macromolecular structure archive. Methods Mol Biol 1607:627–641. doi:10.1007/978-1-4939-7000-1_26 28573592 PMC5823500

[29] Jandova Z, Fast D, Setz M, Pechlaner M, Oostenbrink C. 2018. Saturation mutagenesis by efficient free-energy calculation. J Chem Theory Comput 14:894–904. doi:10.1021/acs.jctc.7b01099 29262673 PMC5813279

[30] Buß O, Rudat J, Ochsenreither K. 2018. FoldX as protein engineering tool: better than random based approaches? Comput Struct Biotechnol J 16:25–33. doi:10.1016/j.csbj.2018.01.002 30275935 PMC6158775

[31] Gudmundsson S, Singer-Berk M, Watts NA, Phu W, Goodrich JK, Solomonson M, Genome Aggregation Database Consortium, Rehm HL, MacArthur DG, O’Donnell-Luria A. 2022. Variant interpretation using population databases: lessons from gnomAD. Hum Mutat 43:1012–1030. doi:10.1002/humu.24309 34859531 PMC9160216

[32] Stenson PD, Mort M, Ball EV, Chapman M, Evans K, Azevedo L, Hayden M, Heywood S, Millar DS, Phillips AD, Cooper DN. 2020. The Human Gene Mutation Database (HGMD): optimizing its use in a clinical diagnostic or research setting. Hum Genet 139:1197–1207. doi:10.1007/s00439-020-02199-3 32596782 PMC7497289

[33] Gu Z, Hübschmann D. 2022. Make interactive complex heatmaps in R. Bioinformatics 38:1460–1462. doi:10.1093/bioinformatics/btab806 34864868 PMC8826183

[34] Rosignoli S, Paiardini A. 2022. Boosting the full potential of PyMOL with structural biology plugins. Biomolecules 12:1764. doi:10.3390/biom12121764 36551192 PMC9775141

[35] Xiong H-L, Wu Y-T, Cao J-L, Yang R, Liu Y-X, Ma J, Qiao X-Y, Yao X-Y, Zhang B-H, Zhang Y-L, Hou W-H, Shi Y, Xu J-J, Zhang L, Wang S-J, Fu B-R, Yang T, Ge S-X, Zhang J, Yuan Q, Huang B-Y, Li Z-Y, Zhang T-Y, Xia N-S. 2020. Robust neutralization assay based on SARS-CoV-2 S-protein-bearing vesicular stomatitis virus (VSV) pseudovirus and ACE2-overexpressing BHK21 cells. Emerg Microbes Infect 9:2105–2113. doi:10.1080/22221751.2020.1815589 32893735 PMC7534347

